# The Impact of Surgical Scheduling on Outcomes in Lumbar Laminectomy

**DOI:** 10.7759/cureus.20272

**Published:** 2021-12-08

**Authors:** David Bailey, Morgan Lehman, Kyle Tuohy, Elizabeth Ko, Steven Hatten, Elias Rizk

**Affiliations:** 1 Neurological Surgery, Penn State Health Milton S. Hershey Medical Center, Hershey, USA

**Keywords:** health systems, surgical complications, laminectomy, time of day, surgical outcomes, fatigue

## Abstract

Objective

The purpose of this study was to determine whether surgical scheduling affected patient outcomes following lumbar laminectomy. Physician fatigue caused by prolonged work hours has been shown to worsen outcomes. Previous research has also established a relationship between surgical scheduling and outcomes.

Methods

This was a retrospective chart review of single-level lumbar laminectomy patients at the Penn State Milton S. Hershey Medical Center between 1992 and 2019. Patients who underwent a one-level laminectomy between 1992 and 2019 were included in the study. Patients with procedures defined as complex (>1 level, tumor or abscess removal, discectomy, implant removal) were excluded. The surgical complication rate [cerebrospinal fluid (CSF) leak, 30-day redo, 30-day ED visit, weakness, sensation loss, infection, urinary retention] was compared across surgical start times, day of the week, proximity to a holiday, and procedure length.

Results

Procedures that started between 9:01-11:00 were more likely to have a complication than those between 7:01-9:00 (p=0.04). For every 60-min increase in surgery length, odds of having a complication increased by 2.01 times (p=0.0041). Surgeries that started between 11:01-13:00 had a significantly longer median surgery length than those between 7:01-9:00.

Conclusion

The time of the day when the procedure was started was predictive of worse outcomes following laminectomy. This may be attributed to several factors, including fatigue and staff turnover. Additionally, increased surgical length was predictive of more complications. It remains unclear whether increased surgical time results from correction of noticed errors or a fatigue-related decline in speed and performance. These findings on one-level laminectomy warrant further investigations since they have implications for reducing systemic failures that impact patient outcomes.

## Introduction

Since the publication of the 1999 Institute of Medicine's report, which revealed that 98,000 annual deaths are attributable to medical errors, increased focus has been placed on systemic changes to improve patient safety and outcomes [1]. Surgical errors are of particular concern since it is estimated to contribute to up to two-thirds of adverse events [2,3]. More than half of these errors were preventable, and a majority were determined to be technique-related errors that occurred intraoperatively [2].

Fatigue has been proven to decrease cognitive and motor abilities among clinicians, which leads to a decline in performance that is associated with worse patient and provider outcomes [4]. Significantly, the practice of surgery is not immune to these fatigue-related deficits in cognitive and motor performance. Studies have shown that fatigue levels increase the risk of medical errors in surgical residents compared with well-rested control subjects [5,6]. This association is something that surgeons intrinsically understand, with 33% of surveyed surgeons attributing fatigue to operative errors [7]. Fatigue-related deficits are especially relevant within the context of surgeons' lifestyles, where many surgeons believe that working before or beyond the stipulated hours of 8 am to 6 pm is a standard part of the job [8]. While this schedule may be universally accepted, it is important to consider the impact of surgical scheduling on surgical outcomes.

Previous research findings on this topic have been equivocal. In the emergent and transplant literature, there does not appear to be any difference in outcomes based on the time of day [9-15]. However, the findings of research on non-emergent surgeries across a number of different fields are mixed [16-18]. In light of this, we aimed to further investigate the impact of surgical scheduling on outcomes in non-complex one-level laminectomies.

## Materials and methods

This study utilized a retrospective chart review to analyze outcomes in one-level laminectomy at Penn State Milton S. Hershey Medical Center. Patient information was collected using TriNetX (TriNetX, Cambridge, MA), which is a database that is searchable by patient characteristics. The Institutional Review Board at Penn State approved this study and deemed patient consent unnecessary as the data were de-identified.

Information was obtained on patients who underwent a laminectomy between 1992 and 2019. This included 4,899 patients from both the neurological and orthopedic services who underwent a single-level laminectomy with no previous spinal procedure history. Patients who had complex procedures were excluded. Complexity was defined as follows: >1 level, tumor or abscess removal, discectomy, or implant removal. Based on these criteria, a total of 554 procedures were included in the final analysis.

Chart review collected data, including surgical date, start time, and end time. This information was obtained from operative nursing reports. In cases where start and end times were not provided, the start time was obtained from the "beginning of encounter" field, found in the surgeons' operative report. In the absence of a nursing record, no information was collected on surgical length due to the lack of end time. Covariates included patient name, sex, age, and surgeon details. Data on surgical complications were collected via a review of the operative report, evaluation performed immediately after the surgery, and the 30-day postoperative appointment. Complications included cerebrospinal fluid (CSF) leak/durotomy, repeat procedures, new weakness or sensation loss, postoperative urinary retention, wound infection, 30-day readmission, or 30-day ED visit for complaints related to the procedure. Complications were weighted equally as "yes/no" for each instance.

Variables were summarized with frequency tables or with means, medians, standard deviations, etc., prior to any analysis to assess their distributions. Time of day for surgical start time was defined as either AM/PM or broken down into categories including 7:01-9:00, 9:01-11:00, 11:01-13:00, 13:01-15:00, 15:01-17:00, and 17:01-7:00 with 7:01-9:00 used as the reference category for analysis. Saturday and Sunday were combined for days of the week of surgery. All the individual days of the week were compared against Monday. Surgical dates within one week preceding or following a holiday were compared to those that were not in this range. The holidays included Christmas, New Year’s Day, Martin Luther King Day, Memorial Day, the Fourth of July, Labor Day, and Thanksgiving.

Multivariable logistic regression was used to determine the association of surgery time of day, surgery day of the week, surgery proximity to a holiday, or surgical length with any surgical complications while adjusting for age, MetRx^TM^, level, and surgeon details. The magnitude and direction of any significant associations were quantified with odds ratios (OR), and the fit of the final model was checked using the Hosmer-Lemeshow goodness-of-fit test. Multivariable quantile regression of the median was used to determine any differences in median surgery length between surgical start time categories while adjusting for age, MetRx^TM^, level, and surgeon details. All analyses used a significance level (type I error) of 0.05 and were performed using SAS version 9.4 (SAS Institute, Cary, NC).

## Results

Data from 554 neurosurgical one-level laminectomies were obtained and analyzed. The average age of the cohort was 63.25 years, and 53% were male. Of the procedures performed, 548 (98.9%) occurred on weekdays. Day of the week breakdown was as follows: 105 (18.9%) on Monday, 174 (31.4%) on Tuesday, 146 (26.3%) on Wednesday, 81 (14.6%) on Thursday, and 42 (7.6%) on Friday. The other six (1.08%) included five (0.9%) on Saturday and one (0.18%) on Sunday. There were 141 (25.45%) surgeries that occurred within one week of a holiday when work was typically excused. This included Christmas, New Year's Day, Martin Luther King Day, Memorial Day, the 4th of July, Labor Day, and Thanksgiving. There was a total of 219 (39.6%) procedures that utilized the MetRx^TM^ system. A total of 216 (38.99%) procedures resulted in complications; 459 (84%) procedures occurred before 12 pm, with a majority (263, 48.17%) occurring in the 7:01-9:00 timeframe and then decreasing across the rest of the day (Table 1).

**Table 1:** Demographic information of patients undergoing single-level lumbar laminectomy between 1992 and 2019 *One patient was not included in this parameter. ^†^Some patients had more than one complication, so the numbers for each individual complication do not add up to the value for "Any complication". ^‡^82 patients were not included in this characteristic because they did not have surgery length information available in their records. ^§^Eight patients were not included in this characteristic because they did not have the time of day information available in their records CSF: cerebrospinal fluid; ED: emergency department; SD: standard deviation

Operative characteristics	
Age (years), mean ± SD	63.25 ± 13.18
Sex, n (%)	
Male	294 (53.07)
Female	260 (46.93)
Laminectomy level, n (%)	
L1	5 (0.90)
L2	47 (8.48)
L3	109 (19.68)
L4	336 (60.65)
L5	57 (10.29)
METRx^TM^ system used, n (%)	219 (39.60)
Weekday, n (%)	548 (98.92)
Monday	105 (18.95)
Tuesday	174 (31.41)
Wednesday	146 (26.35)
Thursday	81 (14.62)
Friday	42 (7.58)
Weekend, n (%)	6 (1.08)
Saturday	5 (0.90)
Sunday	1 (0.18)
Any complication, n (%)^†^	216 (38.99)
CSF leak	79 (14.26)
Operation redone	93 (16.82)
Weakness	16 (2.90)
Sensation loss	20 (3.63)
Urinary retention	43 (7.79)
Infection	8 (1.45)
30-day readmission	12 (2.17)
ED visit	28 (5.06)
Surgery length (minutes) mean ± SD^‡^	77.96 ± 29.64
Scheduling characteristics	
12-hour time period, n (%)^^^	
AM	459 (84.07)
PM	87 (15.93)
Time of day, n (%)^§^	
7:01-09:00	263 (48.17)
9:01-11:00	98 (17.95)
11:01-13:00	93 (17.03)
13:01-15:00	61 (11.17)
15:01-17:00	21 (3.85)
17:01-07:00	10 (1.83)
1 week before or after a holiday, n (%)	141 (25.45)

There was no significant difference in complication rates between morning and afternoon procedures (OR: 0.71, 95% CI: 0.436-1.158, p=0.17). Procedures occurring between 9:01-11:00 were more likely to have complications than those that occurred between 7:01-9:00 (OR: 1.69, 95% CI: 1.018-2.809, p=0.04). All other time categories had comparable complication rates (Table 2).

**Table 2:** Multivariate logistic regression to determine the association between surgical start time and complications** *Significant (p<0.05). **Analysis adjusted for age, MetRx^TM^, spinal level, and surgeon details

Procedure start time and surgical complications				
AM vs. PM start times				
Categories	N (%)	Number of procedures with complications, n (%)	Odds ratio of any complication occurring (95% CI)	P-value
AM	458 (84.04)	173 (37.55)	0.71 (0.44-1.16)	0.17
PM	87 (15.96)	40 (45.98)		
Start times by category				
Surgical start time	Total (% of all procedures)	Procedures with complications, n (%)	Odds ratio, compared to 7:01-9:00 (95% CI)	P-value
7:01-9:00	262 (48.07)	88 (33.59)	-	-
9:01-11:00	98 (17.98)	47 (47.96)	1.69 (1.02-2.81)	0.04^*^
11:01-13:00	93 (17.06)	36 (38.71)	1.26 (0.76-2.10)	0.37
13:01-15:00	61 (11.19)	28 (45.90)	1.60 (0.88-2.88)	0.12
15:01-17:00	21 (3.85)	8 (38.10)	1.19 (0.46-3.11)	0.72
17:01-07:00	10 (1.83)	5 (50.00)	2.12 (0.56-7.97)	0.27

Whether the procedure was performed on the weekend or weekday was not a predictor of worse outcomes (OR: 9.224, 95% CI: 0.996-85.381, p=0.0504). There was no difference in complication rates between specific days of the week, with surgical outcomes remaining similar when comparing Monday to later days (Monday vs. Friday: OR: 0.61, 95% CI: 0.279-1.337, p=0.22). There was no statistically significant relationship between complication rates and proximity to holidays. Procedures performed within seven days of a holiday did not have higher complication rates (OR: 1.24, 95% CI: 0.83-1.85, p=0.29). There was also no individual holiday where proximity within seven days was predictive of worse outcomes (Table 3).

**Table 3:** Multivariate logistic regression to determine the association between day of the week and proximity to holiday with the rate of complications* *Analysis adjusted for age, MetRx^TM^, spinal level, and surgeon details

Day of the procedure and surgical complications				
Day of the week				
Categories	N (%)	Number of procedures with complications, n (%)	Odds ratio of any complication occurring (95% CI)	P-value
Weekday	547 (98.92)	210 (38.39)	9.22 (1.00-85.38)	0.05
Weekend	6 (1.08)	5 (83.33)		
Day of the week	Total (% of all procedures)	Procedures with complications, n (%)	Odds ratio, compared to Monday (95% CI)	P-value
Monday	105 (18.99)	47 (44.76)		-
Tuesday	174 (31.46)	66 (37.93)	0.67 (0.37-1.22)	0.19
Wednesday	146 (26.40)	51 (34.93)	0.56 (0.30-1.04)	0.07
Thursday	80 (14.47)	32 (40.00)	0.62 (0.29-1.34)	0.22
Friday	42 (7.59)	14 (33.33)	0.61 (0.28-1.34)	0.22
Saturday/Sunday	6 (1.08)	5 (83.33)	6.41 (0.67-61.39)	0.11
Proximity to a holiday				
Categories	N (%)	Number of procedures with complications, n (%)	Odds Ratio of any complication occurring (95% CI)	P-value
±7 days of holiday	141 (25.45)	60 (42.55)	1.24 (0.83-1.85)	0.29
Not within 7 days of Holiday	412 (74.50)	155 (37.62)		

There was a significant relationship between the surgery length and having complications (OR: 2.01, 95% CI: 1.248-3.239, p=0.0041). For every 60-minute increase in surgery time, the odds of complication increased by 2.01 times. Procedure start time was not related to the overall length of surgeries, although PM surgeries had a higher median surgery length versus AM surgeries (83 vs. 71 minutes). Surgeries that started between 11:01-13:00 did have a significantly longer median surgery length than surgeries that started between 7:01-9:00 (Table 4).

**Table 4:** Multivariable quantile regression of the median to determine any differences in median surgery length between surgical start time categories *Significant (p<0.05). **Analysis was adjusted for age, MetRx^TM^, spinal level, and surgeon details. ^†^Odds ratio for every 60-minute increase in surgical time

Surgical length and surgical complications		
Variable	Odds ratio of any complication occurring (95% CI)	P-value
Surgical length	2.01 (1.25-3.24)^†^	0.004^*^
Relationship between surgical start time and surgical length		
AM vs. PM start times	Difference of medians (minutes)	Pr >|t|
AM	-7.0 (-14.77, 0.77)	0.06
PM	-	
Start times by category		
Surgical start time	Difference of medians (minutes)	Pr >|t|
7:01-09:00	-	-
9:01-11:00	1.0 (-6.41, 8.41)	0.79
11:01-13:00	7.0 (1.44, 12.56)	0.001^*^
13:01-15:00	7.0 (-2.11, 16.11)	0.13
15:01-17:00	9.0 (-6.76, 24.76)	0.26
17:01-07:00	35.0 (-47.13, 117.13)	0.40

## Discussion

The relationship between fatigue and performance-related errors has been well established in the hospital setting, with worsening fatigue predictive of a decline in physician performance [5,6,19-22]. Fatigue worsens psychomotor performance in anesthesiologists and impairs the speed and accuracy of surgeons and surgical residents [5,19,20]. Multiple reports have demonstrated that the risks of making an error increased when shifts were longer than 12 hours, the provider was working overtime or working more than 40 hours that week, or had frequent 24-hour shifts [21-22]. Surgical residents during post-call take a longer time on the job and commit more errors in surgical tasks when compared to well-rested controls when studied via simulation [6,23].

This knowledge has led to several systematic changes to reduce physician fatigue. An example is a work-hour limitation set by the Accreditation Council for Graduate Medical Education (ACGME) to limit residents' clinical hours per week. Overall, these restrictions have had mixed success. Surveyed residents report feeling fatigued 48% of the time during their shift and impaired 27% of the time [5]. Criticism of these reforms cites that hour limitations are not adequately preparing doctors since an important part of the training involves overcoming fatigue. Analysis of the efficacy of work-hour restrictions demonstrated no difference in patient mortality, readmissions, or care costs among residents following work-hour reforms [24]. However, it remains true that physicians must be prepared to work impressively long hours in their future practice. This often results from combining clinical practice with other pursuits, both professionally and personally. Overextension of self is almost an expectation from physicians that has become normalized with a "work until the work is done" attitude.

It is pertinent to ask whether this attitude has created inequity in surgical outcomes due to surgical scheduling and subsequent fatigue-related deficits. Kelz et al. have demonstrated that a later start time in non-emergent general surgery procedures leads to higher morbidity and mortality [16]. This includes a higher risk of deep wound infection and wound dehiscence, both of which may be attributed to technical errors. Research by Komen et al. has shown a higher likelihood of anastomotic leak following colorectal repair even when controlling for urgency [25]. Li et al. have demonstrated that patients suffer increased morbidity, more prolonged operations, and higher cost in total knee replacement when the case is performed fourth in the order or later [17]. Thomas et al. have demonstrated that an increasing number of hours worked was predictive of worse pulmonary lobectomy outcomes [26].

In neurosurgery specifically, Linzey et al. have demonstrated that more surgical complications occur as the day progresses across a wide range of neurosurgical procedures when controlling for emergent vs. non-emergent procedures [27]. In addition, Chern et al. have demonstrated that CSF shunt surgeries occurring later in the day are more likely to require revision surgery [28]. They found that operations starting after 3 pm were more likely to need a reoperation on readmission. Alternatively, Lu et al. have demonstrated that afternoon liver resections did not result in worse outcomes compared to those in the morning [18]. This null association was attributed to many factors, including the surgeons' skill, judgment, and self-regulation, which allowed them to overcome fatigue-related deficits. Also, the trauma and transplant literature generally does not reflect the pattern of worsening fatigue causing increased errors [9-15]. The discrepancy in the literature makes the impact of the time of day on surgical outcomes unclear.

Our research demonstrates a significant increase in complication rates in the 9:01-11:00 period relative to 07:01-9:00. Many etiologies may explain the potential decremental relationship between the time of day and surgical outcomes. One very likely mechanism is the time of day representing the level of provider fatigue, supported by worsening outcomes depending on hours worked and case order [17]. Another explanation may be that the 9:01 start times were originally scheduled for 7:01 and were postponed to a later start. Alternatively, it is possible that the disruptive nature of shift change of surgical support staff may be the true underlying etiology [16,18]. These reasons are supported by the null findings in emergent and transplant literature, where the surgical team is specially trained to cope with unusual hours and does not follow the same staffing procedures [14]. Ultimately, our finding of an increased complication rate later in the day further adds to the literature suggesting that surgical start time may impact patient outcomes. However, these findings are not generalizable and warrant further study in a broader range of specialties and procedures.

In addition to the time of day, we also analyzed the impact that day of the week has on surgical outcomes. Previous research has demonstrated that acute admission on the weekend may increase morbidity and mortality [29]. An analysis by Freemantle et al. has demonstrated an increased risk of mortality on the weekends even when controlling for the severity of illness. Their results demonstrated that 11,000 more people die within 30 days when admitted between Friday and Monday relative to the rest of the week [30]. This "weekend effect" is attributable to differences in staffing patterns and shortcomings in resource allocation.

Data for elective surgery also demonstrates this weekend effect. Aylin et al. have demonstrated a higher risk of death for elective procedures performed later in the week and the weekend [31]. O'Leary et al. have shown that elective admission on the weekend for patients who have surgery resulted in increased mortality relative to those not admitted on the weekend, regardless of whether the surgery was performed on the weekend or subsequent weekday [32]. Zare et al. have demonstrated that patients admitted to regular hospital floors after non-emergent major surgery had increased mortality if the surgery was performed on Friday versus those performed Monday through Wednesday [33]. In neurosurgery, recent research has demonstrated that elective spine surgery on Thursday and Friday has a longer average stay than those procedures occurring on Monday and Tuesday despite no difference in complication rates [34]. The authors suggest that strategic surgical scheduling may be an effective strategy for decreasing health system costs.

In addition to this weekend effect, previous research has demonstrated that patients have a 4x increase in mortality risk within five days of surgery and 2x within 30 days of surgery if their procedure occurs during a holiday period. This research by Foss and Kehlet, involving a population of acute surgical patients, suggests a significant decrease in the quality of care during the holiday period [35]. The authors attributed their findings to changes in staffing patterns perioperatively, with a significant reduction in nursing and physical therapy staff during the holiday time. Their results support additional research suggesting an increased mortality risk when the nurse-to-patient ratio decreases [36]. The results of our study demonstrate neither a weekend nor a holiday effect. There was no significant difference in outcomes based on the day of the week or if the surgery occurred during the seven days before and after a holiday. This null finding may be due to the limited intensity of perioperative needs surrounding a one-level laminectomy. Additionally, the low number of weekend procedures in our analysis may not have had adequate power to detect any significant difference when comparing weekend versus weekday procedures.

Our analysis did find a significant relationship between the rate of complications and surgical length. As operative time increased, so too did the rate of complications. This finding supports previous research demonstrating a relationship between increased complication rate and surgical length [37,38]. The significance of this finding is unclear. It is possible that as operations dragged on, surgeons became more fatigued and committed more errors. Alternatively, the increased surgical length may be related to recognition and subsequent repair of operative errors, which may be unrelated to fatigue, thereby prolonging the operative time. Previous research has demonstrated several other factors besides fatigue that predict complications in patients undergoing one-level laminectomy [39-41]. This includes older age, surgeon experience level, comorbidities, and a history of previous surgery. Significantly, our analysis controls for these covariates.

This finding is important in the context of the additional association between surgical start time and surgical length, with longer median surgery length reported later in the day. This contradicts previous research, which demonstrated that later start times are associated with shorter procedures [16,27]. Linzey et al., who demonstrated that shorter procedures occur later in the day and that shorter procedures have a higher rate of complications, theorized that the shorter surgical length later in the day was due to surgeon fatigue and subsequent hastiness [27]. Our own analysis does not support this conclusion, demonstrating that longer procedures produce more complications and that surgical length increases with later start times.

The study was conducted at a single center (Penn State Hershey Medical Center) with a select group of surgeons and may not be generalizable to another institution’s practice or any individual surgeon. The retrospective nature of this analysis may also limit our understanding of this surgical population. Limitations include misclassification of patients and incomplete documentation as a result of the retrospective design. Incomplete documentation includes a number of instances where data related to total operative time could not be collected and these patients were subsequently excluded from the relevant analysis. Additionally, our analysis did not include a clear understanding of why some procedures started "after-hours." There was no way to deduce surgeons’ prior actions, including previous procedures and call schedules that may amplify fatigue. Future studies are required to overcome these limitations and further understand the impact that the time of day has on surgical complications.

## Conclusions

With the exception of the 9:00-11:00 time period, our analysis demonstrated no significant difference in outcomes based on the time of day in one-level laminectomy procedures. This finding further contributes to the literature on the impact of fatigue and physician performance. Additionally, there was a significant association between start time and median surgical length as well as surgical length and complication rate, with complications increasing 2x with every hour of case duration. It is unclear whether this increased complication rate is due to surgeon fatigue or due to complications necessitating increased operative time. Further analysis is required to gain deeper insights into the impact that the time of day has on surgical outcomes, which may have implications for quality improvement in health systems.

## References

[1] Institute of Medicine (2000). To Err Is Human. To Err Is Human: Building a Safer Health System.

[2] Gawande AA, Thomas EJ, Zinner MJ, Brennan TA (1999). The incidence and nature of surgical adverse events in Colorado and Utah in 1992. Surgery.

[3] Leape LL, Brennan TA, Laird N (1991). The nature of adverse events in hospitalized patients. Results of the Harvard Medical Practice Study II. N Engl J Med.

[4] Wright MC, Phillips-Bute B, Mark JB, Stafford-Smith M, Grichnik KP, Andregg BC, Taekman JM (2006). Time of day effects on the incidence of anesthetic adverse events. Qual Saf Health Care.

[5] McCormick F, Kadzielski J, Landrigan CP, Evans B, Herndon JH, Rubash HE (2012). Surgeon fatigue: a prospective analysis of the incidence, risk, and intervals of predicted fatigue-related impairment in residents. Arch Surg.

[6] Taffinder NJ, McManus IC, Gul Y, Russell RC, Darzi A (1998). Effect of sleep deprivation on surgeons' dexterity on laparoscopy simulator. Lancet.

[7] Gawande AA, Zinner MJ, Studdert DM, Brennan TA (2003). Analysis of errors reported by surgeons at three teaching hospitals. Surgery.

[8] (2021). Who operates when? II. https://www.ncepod.org.uk/2003wow.html.

[9] Saleem MA, Kannam H, Aronow WS, Weiss MB, Kalapatapu K, Pucillo AL, Monsen CE (2004). The effects of off-normal hours, age, and gender for coronary angioplasty on hospital mortality in patients undergoing coronary angioplasty for acute myocardial infarction. Am J Cardiol.

[10] Gabriel RA, A'Court AM, Schmidt UH, Dutton RP, Urman RD (2018). Time of day is not associated with increased rates of mortality in emergency surgery: an analysis of 49,196 surgical procedures. J Clin Anesth.

[11] Sugünes N, Bichmann A, Biernath N (2019). Analysis of the effects of day-time vs night-time surgery on renal transplant patient outcomes. J Clin Med.

[12] Zahn R, Schiele R, Seidl K (1999). Daytime and nighttime differences in patterns of performance of primary angioplasty in the treatment of patients with acute myocardial infarction. Maximal Individual Therapy in Acute Myocardial Infarction (MITRA) Study Group. Am Heart J.

[13] Zafar SN, Libuit L, Hashmi ZG (2015). The sleepy surgeon: does night-time surgery for trauma affect mortality outcomes?. Am J Surg.

[14] George TJ, Arnaoutakis GJ, Merlo CA, Kemp CD, Baumgartner WA, Conte JV, Shah AS (2011). Association of operative time of day with outcomes after thoracic organ transplant. JAMA.

[15] Lonze BE, Parsikia A, Feyssa EL (2010). Operative start times and complications after liver transplantation. Am J Transplant.

[16] Kelz RR, Freeman KM, Hosokawa PW (2008). Time of day is associated with postoperative morbidity: an analysis of the national surgical quality improvement program data. Ann Surg.

[17] Li X, Zhang Q, Dong J, Zhang G, Chai W, Chen J (2018). Impact of surgical case order on peri-operative outcomes for total joint arthroplasty. Int Orthop.

[18] Lu Q, Shen Y, Zhang J (2017). Operation start times and postoperative morbidity from liver resection: a propensity score matching analysis. World J Surg.

[19] Howard SK, Gaba DM, Smith BE, Weinger MB, Herndon C, Keshavacharya S, Rosekind MR (2003). Simulation study of rested versus sleep-deprived anesthesiologists. Anesthesiology.

[20] Grantcharov TP, Bardram L, Funch-Jensen P, Rosenberg J (2001). Laparoscopic performance after one night on call in a surgical department: prospective study. BMJ.

[21] Rogers AE, Hwang WT, Scott LD, Aiken LH, Dinges DF (2004). The working hours of hospital staff nurses and patient safety. Health Aff (Millwood).

[22] Landrigan CP, Rothschild JM, Cronin JW (2004). Effect of reducing interns' work hours on serious medical errors in intensive care units. N Engl J Med.

[23] Eastridge BJ, Hamilton EC, O'Keefe GE (2003). Effect of sleep deprivation on the performance of simulated laparoscopic surgical skill. Am J Surg.

[24] Jena AB, Farid M, Blumenthal D, Bhattacharya J (2019). Association of residency work hour reform with long term quality and costs of care of US physicians: observational study. BMJ.

[25] Komen N, Dijk JW, Lalmahomed Z (2009). After-hours colorectal surgery: a risk factor for anastomotic leakage. Int J Colorectal Dis.

[26] Thomas M, Allen MS, Wigle DA, Shen KR, Cassivi SD, Nichols FC 3rd, Deschamps C (2012). Does surgeon workload per day affect outcomes after pulmonary lobectomies?. Ann Thorac Surg.

[27] Linzey JR, Burke JF, Sabbagh MA, Sullivan S, Thompson BG, Muraszko KM, Pandey AS (2018). The effect of surgical start time on complications associated with neurological surgeries. Neurosurgery.

[28] Chern JJ, Bookland M, Tejedor-Sojo J, Riley J, Shoja MM, Tubbs RS, Reisner A (2014). Return to system within 30 days of discharge following pediatric shunt surgery. J Neurosurg Pediatr.

[29] Bell CM, Redelmeier DA (2001). Mortality among patients admitted to hospitals on weekends as compared with weekdays. N Engl J Med.

[30] Freemantle N, Ray D, McNulty D, Rosser D, Bennett S, Keogh BE, Pagano D (2015). Increased mortality associated with weekend hospital admission: a case for expanded seven day services?. BMJ.

[31] Aylin P, Alexandrescu R, Jen MH, Mayer EK, Bottle A (2013). Day of week of procedure and 30 day mortality for elective surgery: retrospective analysis of hospital episode statistics. BMJ.

[32] O'Leary JD, Wunsch H, Leo AM, Levin D, Siddiqui A, Crawford MW (2019). Hospital admission on weekends for patients who have surgery and 30-day mortality in Ontario, Canada: a matched cohort study. PLoS Med.

[33] Zare MM, Itani KM, Schifftner TL, Henderson WG, Khuri SF (2007). Mortality after nonemergent major surgery performed on Friday versus Monday through Wednesday. Ann Surg.

[34] Salas-Vega S, Chakravarthy VB, Winkelman RD (2021). Late-week surgery and discharge to specialty care associated with higher costs and longer lengths of stay after elective lumbar laminectomy. J Neurosurg Spine.

[35] Foss NB, Kehlet H (2006). Short-term mortality in hip fracture patients admitted during weekends and holidays. Br J Anaesth.

[36] Aiken LH, Clarke SP, Sloane DM, Sochalski J, Silber JH (2002). Hospital nurse staffing and patient mortality, nurse burnout, and job dissatisfaction. JAMA.

[37] Runkel NS, Schlag P, Schwarz V, Herfarth C (1991). Outcome after emergency surgery for cancer of the large intestine. Br J Surg.

[38] Korinek AM (1997). Risk factors for neurosurgical site infections after craniotomy: a prospective multicenter study of 2944 patients. The French Study Group of Neurosurgical Infections, the SEHP, and the C-CLIN Paris-Nord. Service Epidémiologie Hygiène et Prévention. Neurosurgery.

[39] Sin AH, Caldito G, Smith D, Rashidi M, Willis B, Nanda A (2006). Predictive factors for dural tear and cerebrospinal fluid leakage in patients undergoing lumbar surgery. J Neurosurg Spine.

[40] Smorgick Y, Baker KC, Herkowitz H (2015). Predisposing factors for dural tear in patients undergoing lumbar spine surgery. J Neurosurg Spine.

[41] Li G, Patil CG, Lad SP, Ho C, Tian W, Boakye M (2008). Effects of age and comorbidities on complication rates and adverse outcomes after lumbar laminectomy in elderly patients. Spine (Phila Pa 1976).

